# Accuracy of pedicle screw placement in the thoracic and lumbosacral spines using O-arm-based navigation versus conventional freehand technique

**DOI:** 10.1186/s41016-019-0154-y

**Published:** 2019-03-04

**Authors:** Linkai Jing, Zhenze Wang, Zhenxing Sun, Huifang Zhang, James Wang, Guihuai Wang

**Affiliations:** 1 School of Clinical Medicine, Tsinghua University, Beijing, 100084 China; 2 Department of Neurosurgery, Beijing Tsinghua Changgung Hospital, School of Clinical Medicine, Tsinghua University, Beijing, 102218 China; 3 Department of Neurosurgery, Haicheng Zhenggu Hospital, Anshan City, 114200 Liaoning China

**Keywords:** Accuracy, Freehand, Navigation, O-arm, Pedicle screw

## Abstract

**Background:**

The accuracy and safety of pedicle screw insertion was markedly improved with the introduction of intraoperative three-dimensional navigation system during the last decade. This study aimed to evaluate the accuracy of pedicle screw placement using O-arm-based navigation system versus conventional freehand technique.

**Methods:**

We reviewed the accuracy of 341 thoracic (*n* = 173) and lumbosacral (*n* = 168) pedicle screws placed in 60 consecutive patients using either O-arm-based navigation or freehand technique in the Department of Neurosurgery of Beijing Tsinghua Changgung Hospital between January 2015 and June 2018. Patient-specific characteristics, treatment-related characteristics, and screw-specific accuracy were analyzed. The accuracy of pedicle screw placement was measured by Gertzbein-Robbins scale and screw grades A and B were clinically acceptable.

**Results:**

One hundred ninety-one screws were inserted in the O-arm-based navigation group and 150 in the freehand group. One hundred eighty-three (95.81%) clinically acceptable screws were placed in the navigation group and 135 (90.00%) in the freehand group (*p* = 0.034). Twenty-three (6.74%) screw revisions were performed in the two groups (8 screws in the navigation group and 15 screws in the freehand group) and significant difference was observed in thoracic spine (*p* = 0.018), while no statistical significance was presented in lumbosacral spine (*p* > 0.05). Twenty-four (12.57%) screws in the navigation group and 24 (16.00%) in the freehand group violated the cortex (*p* > 0.05). Medial screw deviation was the most common problem in the two groups.

**Conclusion:**

The O-arm-based navigation exhibits higher accuracy for pedicle screw insertion than the freehand insertion technique.

## Background

Pedicle screw insertion is typically performed to treat a number of spinal conditions, including geneogenous, degenerative, traumatogenic, and neoplastic lesions [1–6]. Accurate insertion of pedicle screws is a crucial step and has a direct effect on the surgical outcomes. Experience-based judgment, however, including that based on anatomic landmarks, tactile feedback, and fluoroscopic images, does not entirely prevent occasional errors. Incorrectly placed screws may result in compromised biomechanics or pedicle cortical breaches, especially medial breaches, leading to poor clinical outcomes [4].

With advances in frame fixation, camera technology, automatic registration, and image manipulation software, computer-assisted navigation surgery is proving extremely useful and reliable during procedures performed on the spine [2, 6, 7]. Such advances allow surgeons to track and monitor the instruments used relative to the patient’s anatomy in real time using markers and appropriate software, thereby allowing intraoperative planning, reducing errors, and improving clinical outcomes.

Following the introduction of the O-arm® and StealthStation system (Medtronic, Minneapolis, MN, USA), we retrospectively reviewed and evaluated the accuracy of pedicle screw insertion using this O-arm-based navigation and compared with conventional freehand technique.

## Methods

The study was approved by the Beijing Tsinghua Changgung Hospital review board. We retrospectively reviewed the accuracy of 341 pedicle screws in 60 consecutive patients who had undergone thoracic and lumbosacral instrumented procedures at our hospital between January 2015 and June 2018. The patients were performed thoracic and lumbosacral instrumented surgery (senior doctors: GW and JW) subsequent to a tumor, a traumatic injury, or a degenerative disease. For patient with a tumor, pedicle screw was inserted when the tumor disrupts the spinal stability. They were divided into two groups according to whether using O-arm navigation or not: the navigation group (35 patients, 191 screws) and the freehand group (25 patients, 150 screws).

**Fig. 1:**
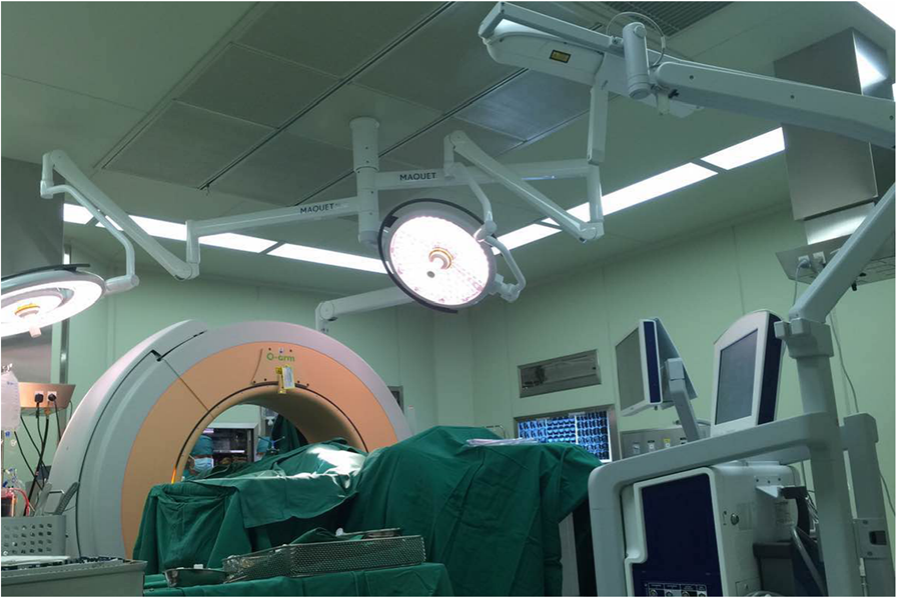
The O-arm imaging system and StealthStation navigation

**Fig. 2:**
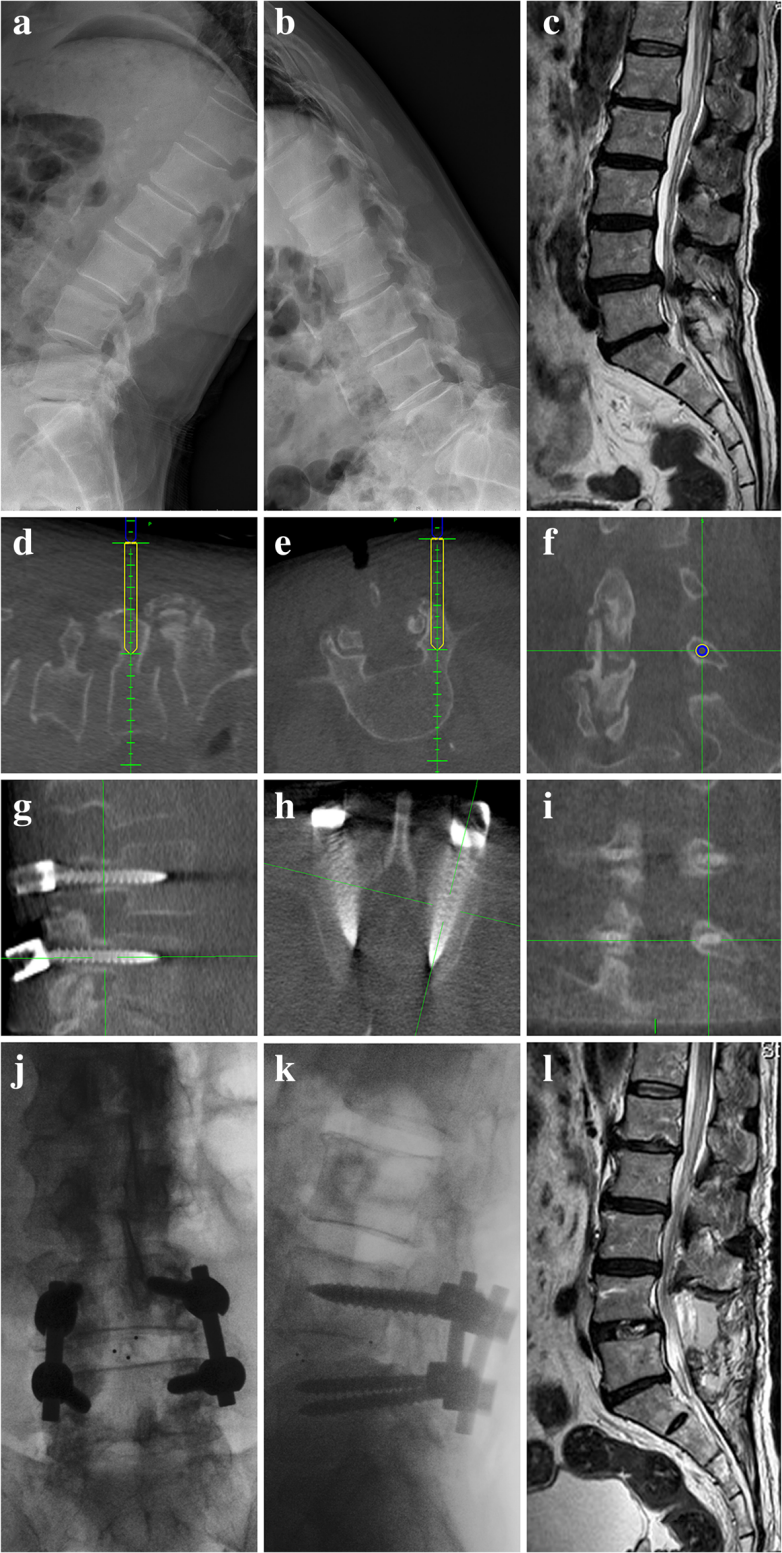
A 59-year-old woman presented with lumbar and lower extremities pain for 3 years. **a**–**c** Preoperative spinal radiography and magnetic resonance imaging (MRI) showed lumbar spondylolisthesis and disc herniation. **d**–**f** Images of the navigation screen. **g**–**i** Images of the O-arm screen obtained from the second intraoperative O-arm scan. **j**, **k** Images of the O-arm screen obtained from the third intraoperative O-arm scan. **l** Postoperative MRI

Patients underwent general anesthesia and then were placed prone position on a Jackson table. Neurosurgeons completed the tumor exposure and resection, as well as decompression, as necessary. For patients with O-arm-navigated pedicle screw insertion, the reference frame was attached to a spinous process, and the O-arm was positioned to obtain three-dimensional (3D) images, which were transferred to the StealthStation navigation system for automatic registration (Fig. 1). The navigation plan was conducted by senior doctors (GW and JW), and the image-guided probe was used to confirm the entry point and orientation of pedicle screws (Fig. 2d–f). The pedicles were cannulated, and the trajectories were visualized on sagittal, axial, and coronal images at the StealthStation. The appropriate screw was selected and inserted. For patients undergoing freehand pedicle screw insertion, pedicles were manually cannulated and tapped according to the surgeon’s experience and the anatomical landmarks.

After appropriate-size screws were placed, the intraoperative O-arm scan with sagittal, axial, and coronal reconstructions was performed to evaluate the positions of the screws in two groups (Fig. 2g–i). Screw position was judged based on the immediately postoperative O-arm images in those three plans. The accuracy of screw insertion was evaluated according to the Gertzbein-Robbins scale grades A–E, where A = no pedicle breach; B = ≤ 2 mm pedicle breach; C = ≤ 4 mm pedicle breach; D = ≤ 6 mm pedicle breach; E = >6 mm pedicle breach [8]. Only screw grades A and B were “clinically acceptable,” which did not need to be revised. For all grade B–E screws, the deviation (cranial, caudal, medial, or lateral) was recorded. An intraoperative 3D O-arm scan was then obtained to confirm the final pedicle screw position when screw revision was required.

Once proper insertion of screws was confirmed, two rods were connected (Fig. 2j, k). Microsurgical procedures in patients with a spinal cord tumor were performed under monitoring of somatosensory evoked potentials and motor evoked potentials. Postoperative CT scans were not obtained given the concerns about the accuracy of the intraoperative O-arm scan, and the additional radiation exposure and cost for the patients.

Data were collected and are summarized in Table 1, including patient-specific characteristics (age, sex, body mass index, and diagnosis), treatment-related data (procedure type, number and distribution of screws, mean time required for screw insertion, blood loss, length of postoperative hospital stay, and radiation dose per screw), and screw-specific accuracy (Gertzbein-Robbins scale grade, screw deviation). Pedicle screw-related complications included neurological deficits (nerve root deficit, cauda equina deficit, spinal cord deficit), infection, vascular and visceral injuries, and cerebrospinal fluid leakage.

**Table 1:** Summary of patient-specific and treatment-related characteristics and screw-specific accuracy

Variables*	Total (*n* = 60)	Navigation group (*n* = 35)	Freehand group (*n* = 25)	*p* value (two-tailed)
Patient-specific characteristics				
Age, years	54.07 (17.93)	54.80 (17.63)	53.04 (18.66)	0.711
Sex, male/female	30/30	17/18	13/12	0.793
Body mass index, kg/m^2^	24.46 (3.98)	24.73 (3.27)	24.08 (4.85)	0.559
Diagnosis				
Tumor (%)	29 (48.33)	15 (42.86)	14 (56.00)	0.45
Degenerative disease (%)	26 (43.33)	16 (45.71)	10 (40.00)	
Trauma (%)	5 (8.33)	4 (11.43)	1 (4.00)	
Treatment-related characteristics				
No. of screws	341	191	150	
Distribution of screws				
Thoracic (%)	173 (50.73)	86 (45.03)	87 (58.00)	0.017
Lumbosacral (%)	168 (49.27)	105 (54.97)	63 (42.00)	
Surgical time/screw, min	18.83 (8.15)	21.36 (8.85)	15.28 (5.47)	0.002
Blood loss, ml	300 (375)	300 (300)	400 (450)	0.652
Length of postoperative hospital stay, days	16.95 (8.73)	16.71 (6.99)	17.28 (10.87)	0.807
Radiation dose/screw, mSv	1.49 (1.12)	1.69 (1.62)	1.25 (1.15)	0.652
Screw-specific accuracy				
Gertzbein-Robbins scale grade, %				
A and B (%)	318 (93.25)	183 (95.81)	135 (90.00)	0.034
C, D, and E (%)	23 (6.74)	8 (4.19)	15 (10.00)	
Screw deviation				
Cranial (%)	5 (10.42)	3 (12.50)	2 (8.33)	0.249
Caudal (%)	9 (18.75)	7 (29.17)	2 (8.33)	
Medial (%)	18 (37.50)	7 (29.17)	11 (45.83)	
Lateral (%)	16 (33.33)	7 (29.17)	9 (37.50)	

*Comparison between navigation and freehand groups. *p* < 0.05 was considered statistically significant

Statistical analysis was performed with an SPSS 19.0 package (IBM Corp., Armonk, NY, USA), and *p* < 0.05 was regarded as indicating statistical significance. For quantitative data, the one-sample Kolmogorov–Smirnov test was used to test the normality of the distribution, and the independent sample *t* test was used for the approximately normal distributed parameters, with data expressed as means ± SD. The rank-sum test was used for non-normally distributed parameters, with data expressed as the median (quartile). The chi-square test was performed for qualitative data.

## Results

### Patient-specific characteristics

A total of 60 consecutive patients (navigation group 35 patients, freehand group 25 patients) who underwent thoracic and lumbosacral instrumented procedures were retrospectively reviewed between May 2015 and April 2018 (Table 1). Among them, 29 (48.33%) operations were performed for tumor, 26 (43.33%) subsequent to degenerative pathology, and 5 (8.33%) for trauma. Patient-specific characteristics (age, sex, and body mass index) showed no significant differences between the two groups (*p* > 0.05) (Table 1).

### Treatment-related characteristics

A total of 341 thoracic (*n* = 173) and lumbosacral (*n* = 168) pedicle screws were inserted in these patients, with 191 screws inserted in the navigation group and 150 screws in the freehand group. Thoracic pedicle screws were more placed in the freehand group than that in the navigation group (87/150 vs. 86/191, *p* = 0.017). The mean time to accomplish each screw insertion was 18.83 ± 8.15 min (navigation group 21.36 ± 8.85 min, freehand group 15.28 ± 5.47 min; *p* = 0.002). The mean blood loss during surgery was 300 ± 375 ml (navigation group 300 ± 300 ml, freehand group 300 ± 450 ml; *p* > 0.05). The mean length of postoperative hospital stay was 16.95 ± 8.73 days (navigation group 16.71 ± 6.99 days, freehand group 17.28 ± 10.87 days; *p* > 0.05). The mean radiation dose of each screw insertion was 1.49 ± 1.12 mSv (navigation group 1.69 ± 1.62 mSv, freehand group 1.25 ± 1.15 mSv; *p* > 0.05). No pedicle screw-related complications occurred in both groups.

### Screw-specific accuracy

Based on confirmatory O-arm scans, 24 (12.57%) screws in navigation group and 24 (16.00%) screws in freehand group were observed cortex violation (grade B to E), with no significant difference between the two groups (*p* > 0.05). Specifically, medial screw deviation was the most common problem: navigation group, 7 screws (29.17%), freehand group, 11 screws (45.83%). According to Gertzbein-Robbins scale grade, accuracy of screw placement was considered clinically acceptable for 183 (95.81%) screws in the navigation group and 135 (90.00%) screws in the freehand group (*p* = 0.034). A total of 23 (6.74%) screw revisions were performed in both groups: 8 (4.19%) screws in the navigation group and 15 (10.00%) screws in the freehand group. We further analyzed the difference of distribution for revised screws and found that significant difference was observed in thoracic group (2 of 86 screws in navigation group and 10 of 87 screws in freehand group; *p* = 0.018) and no statistical significance in lumbosacral group (*p* > 0.05).

## Discussion

Recently, the O-arm multi-dimensional surgical imaging system combined with StealthStation navigation has been widely used for transpedicular screw fixation during spine surgery. The system offers real-time 3D reconstructive images to guide insertion of pedicle screws with a radiation-free working environment for the surgeons and their teams.

### Accuracy of pedicle screw placement

Previous studies demonstrated that the O-arm imaging system combined with the StealthStation navigation system could markedly increase the accuracy of pedicle screw placement and clinical outcomes [2, 3, 5, 6, 9–13]. Rivkin et al. [6] performed a retrospective review of 266 patients who underwent thoracolumbar pedicle screw fixation utilizing the O-arm-guided navigation system and concluded that the overall breach rate was 5.3% (87/1651), including 6.6% thoracic pedicle screws and 5.1% lumbar pedicle screws. Van et al. [5] evaluated the accuracy of 1922 pedicle screws placement using O-arm-guided navigation in an international, multicenter, prospective, clinical study. They found that only 2.5% of the pedicle screws were poorly placed, with 1.8% of the screws classified “unacceptable,” which were revised during the same procedure. Xiao et al. [2] retrospectively investigated clinical outcomes of 1208 patients who underwent open thoracolumbar spinal fusion (614 procedures performed using O-arm-guided navigation, 356 using the freehand technique, 238 using fluoroscopic guidance). Patients experienced shorter hospital stays, lower rates of screw misplacement, and reoperation when using O-arm-assisted navigation technique. Similar to previous reports, our study found that 183 (95.81%) and 135 (90.00%) screws were inserted with clinically acceptable accuracy in the navigation and freehand groups, respectively, with the difference between the groups reaching statistical significance. In addition, revised screws of the thoracic spine in the freehand group were more than that in the navigation group, which may be attributed to the smaller pedicles, particularly between the T4 and T6 levels. These anatomical differences significantly add to the complexity of treatment in thoracic spine particularly when treating complex scoliosis.

Even with the improved precision of pedicle screw placement using O-arm-assisted navigation, there were still eight screws in our study that were not optimally placed. The learning curve associated with O-arm-guided navigation might be the reason for screw malposition, especially by novice surgeons [5, 6, 14]. To become sufficiently skilled to perform this surgery, surgeons with limited O-arm-guided skills had to perform approximately 15–30 operations [6]. Factors associated with incorrect placement of pedicle screws are the presence of congenital scoliosis, neurofibromatosis type I, the concave pedicles, the repaired segment being located three levels away from the reference tracker, and the mid-thoracic spine lesions [1]. In the present study, eight screws in five patients were incorrectly placed because of a spinal tumor or unique thoracic spine anatomy. These cases were revised intraoperatively.

The advances of technology have widened the field of image-guided surgery. Gelalis et al. [3] performed a systematic review of 26 prospective studies regarding the accuracy of 6617 thoracolumbar pedicle screws placement using various techniques. Their results indicated that 69–94% of the screws were fully contained in the pedicle when using freehand technique, 28–85% when using fluoroscopy, 81–92% when using fluoroscopy-based navigation, and 89–100% when using CT navigation. Therefore, CT navigation, or robot-guided surgery, may be the reliable tool to achieve accurate pedicle screw insertion.

### Evaluation of pedicle screw position using the O-arm system

Precise identification of the correct pedicle screw location had been widely based on postoperative thin-cut CT imaging, which has high resolution and good image quality. Nevertheless, 1.35–4.38% of patients required a second surgery for screw revision [6, 7, 15].

Recently, to reduce the need for, and perhaps avoid, reoperative screw revision, surgeons have begun to perform intraoperative O-arm scanning after screw insertion (instead of postoperative CT) to evaluate the position along the screw trajectory with axial, sagittal, and coronal views, thereby allowing revision of suboptimal screw position prior to leaving the operating room [5, 10, 11, 16, 17] (Fig. 2). Larson et al. [17] placed 984 thoracolumbar pedicle screws in 50 pediatric patients and reported that 3.6% (35/984) of the screws required intraoperative revision after obtaining intraoperative scans. None of those patients needed a second surgery. Santos et al. [10] assessed the intraoperative revision and reoperation rates for O-arm-navigated insertion of 988 lumbar pedicle screws in 199 cases. The overall intraoperative revision rate was 4.6%, with none of the patients needing reoperation because of a poorly positioned screw. In this study, we routinely performed intraoperative O-arm scanning after screw placement for all patients to avoid reoperation.

### Radiation exposure using the O-arm system

The radiation dose to the patient was greater with the O-arm-guided navigation technique than with the freehand technique, although the difference was not significant (Table 1). Surgeons and staff, however, had no radiation exposure using intraoperative O-arm-assisted navigation. This system also decreases radiation exposure and cost to patients when evaluating screw accuracy using O-arm images instead of postoperative thin-cut CT. Zhang et al. [18] used identical radiographic techniques and scan length to compare the radiation dose of the O-arm imaging system in the 3D scan acquisition mode with that of the 64-slice CT imaging system. The radiation dose with the 3D O-arm imaging system was approximately half that of the CT scanner. To further decrease the radiation dose of patients, Van et al. [5] compared the surgeon’s confidence in the pedicle screw placement and the actual accuracy as evaluated by the intraoperative O-arm images. The malposition and revision rates were 1.5% and 1.0%, respectively, when the surgeon had complete confidence that the screw was correctly inserted after probing the trajectory, whereas the respective rates were 12.0% and 10.2% when the surgeon doubted that the screw was correctly inserted [5]. Hence, these authors suggested that an additional O-arm scan before wound closure is not necessary when the surgeon is confident of correct screw insertion, while it was of value in case of any doubt. Kassis et al. [19] combined pedicle screw-triggered electromyography with O-arm-based spinal navigation to evaluate the accuracy of 447 screws position in 71 patients. They concluded that redo 3D O-arm scanning after screw insertion could be avoided in 81.7% of patients with negative stimulation but that a 3D scan must be performed in 8.4% of patients with positive stimulation. However, false negative (four patients, 5.6%) and positive (three patients, 4.6%) rates, which are dependent on the chosen stimulation threshold, still influenced the application of such technique. In our institution, the radiation doses for conventional CT scans in the thoracic and lumbosacral spine were 9.56 mSv and 5.99 mSv respectively, whereas with intraoperative 3D O-arm scanning they were 3.83 mSv and 2.16 mSv, respectively. Hence, the radiation dose for patients using O-arm scanning was less than half that when using 64-slice CT scanning, which is consistent with the results of previous studies [5, 18].

### Limitations

Major limitations of our study are its retrospective nature and small patient sample. Although our 60 patients with 341 pedicle screws are a small sample and biases are inevitable, our conclusions showed the possibility of pedicle screw insertion accuracy and clinical outcomes with those procedures. The findings of this research remain to be further verified in larger cohorts from multiple centers. Additionally, the underlying pathologies were heterogeneous and electromyography was not routinely used, which could have affected several parameters and therefore influence the quality of our data and the conclusions derived from the data.

## Conclusions

The O-arm-based navigation does indeed exhibit higher accuracy in pedicle screw insertion than freehand technique.

## Abbreviations

3D: Three-dimensional
CT: Computed tomography
MRI: Magnetic resonance imaging

## References

[1] Jin M, Liu Z, Qiu Y, Yan H, Han X, Zhu Z (2017). Incidence and risk factors for the misplacement of pedicle screws in scoliosis surgery assisted by O-arm navigation-analysis of a large series of one thousand, one hundred and forty five screws. Int Orthop.

[2] Xiao R, Miller JA, Sabharwal NC, Lubelski D, Alentado VJ, Healy AT (2017). Clinical outcomes following spinal fusion using an intraoperative computed tomographic 3D imaging system. J Neurosurg Spine.

[3] Gelalis ID, Paschos NK, Pakos EE, Politis AN, Arnaoutoglou CM, Karageorgos AC (2012). Accuracy of pedicle screw placement: a systematic review of prospective in vivo studies comparing free hand, fluoroscopy guidance and navigation techniques. Eur Spine J.

[4] Samdani AF, Ranade A, Sciubba DM, Cahill PJ, Antonacci MD, Clements DH (2010). Accuracy of free-hand placement of thoracic pedicle screws in adolescent idiopathic scoliosis: how much of a difference does surgeon experience make. Eur Spine J.

[5] Van de Kelft E, Costa F, Van der Planken D, Schils F (2012). A prospective multicenter registry on the accuracy of pedicle screw placement in the thoracic, lumbar, and sacral levels with the use of the O-arm imaging system and StealthStation Navigation. Spine (Phila Pa 1976).

[6] Rivkin MA, Yocom SS (2014). Thoracolumbar instrumentation with CT-guided navigation (O-arm) in 270 consecutive patients: accuracy rates and lessons learned. Neurosurg Focus.

[7] Fichtner J, Hofmann N, Rienmüller A, Buchmann N, Gempt J, Kirschke JS (2018). Revision rate of misplaced pedicle screws of the thoracolumbar spine-comparison of three-dimensional fluoroscopy navigation with freehand placement: a systematic analysis and review of the literature. World Neurosurg.

[8] Gertzbein SD, Robbins SE (1990). Accuracy of pedicular screw placement in vivo. Spine (Phila Pa 1976).

[9] Shin MH, Hur JW, Ryu KS, Park CK (2015). Prospective comparison study between the fluoroscopy-guided and navigation coupled with O-arm-guided pedicle screw placement in the thoracic and lumbosacral spines. J Spinal Disord Tech.

[10] Santos ER, Sembrano JN, Yson SC, Polly DW (2015). Comparison of open and percutaneous lumbar pedicle screw revision rate using 3-D image guidance and intraoperative CT. Orthopedics.

[11] Garber ST, Bisson EF, Schmidt MH (2012). Comparison of three-dimensional fluoroscopy versus postoperative computed tomography for the assessment of accurate screw placement after instrumented spine surgery. Global Spine J.

[12] Silbermann J, Riese F, Allam Y, Reichert T, Koeppert H, Gutberlet M (2011). Computer tomography assessment of pedicle screw placement in lumbar and sacral spine: comparison between free-hand and O-arm based navigation techniques. Eur Spine J.

[13] Jin M, Liu Z, Liu X, Yan H, Han X, Qiu Y (2016). Does intraoperative navigation improve the accuracy of pedicle screw placement in the apical region of dystrophic scoliosis secondary to neurofibromatosis type I: comparison between O-arm navigation and free-hand technique. Eur Spine J.

[14] Ryang YM, Villard J, Obermüller T, Friedrich B, Wolf P, Gempt J (2015). Learning curve of 3D fluoroscopy image-guided pedicle screw placement in the thoracolumbar spine. Spine J.

[15] Samdani AF, Belin EJ, Bennett JT, Pahys JM, Marks MC, Miyanji F (2013). Unplanned return to the operating room in patients with adolescent idiopathic scoliosis: are we doing better with pedicle screws. Spine (Phila Pa 1976).

[16] Tormenti MJ, Kostov DB, Gardner PA, Kanter AS, Spiro RM, Okonkwo DO (2010). Intraoperative computed tomography image-guided navigation for posterior thoracolumbar spinal instrumentation in spinal deformity surgery. Neurosurg Focus.

[17] Larson AN, Santos ER, Polly DW, Ledonio CG, Sembrano JN, Mielke CH (2012). Pediatric pedicle screw placement using intraoperative computed tomography and 3-dimensional image-guided navigation. Spine (Phila Pa 1976).

[18] Zhang J, Weir V, Fajardo L, Lin J, Hsiung H, Ritenour ER (2009). Dosimetric characterization of a cone-beam O-arm imaging system. J Xray Sci Technol.

[19] Kassis SZ, Abukwedar LK, Msaddi AK, Majer CN, Othman W (2016). Combining pedicle screw stimulation with spinal navigation, a protocol to maximize the safety of neural elements and minimize radiation exposure in thoracolumbar spine instrumentation. Eur Spine J.

